# The difference in latent tuberculosis prevalence among medical personnel in negative and non-negative pressure TB isolation ward at Kandou General Hospital, Manado

**DOI:** 10.1017/ash.2025.103

**Published:** 2025-09-03

**Authors:** V Darryl, A Nugroho, P Harijanto

**Affiliations:** Department of Infectious Disease and Tropical Medicine Kandou General Hospital

**Keywords:** Latent tuberculosis, health care workers, negative pressure room

## Abstract

**Background:** Negative-pressure isolation room was considered the standard for Tuberculosis (TB) isolation ward, but it tends to be high in cost and maintenance. Alternatively natural ventilation with the combination of mechanical ventilation (exhaust fan) room were more commonly used in resource-limited settings. However, its efficacy to prevent TB to the medical staffs are unknown. **Objective:** To compare the prevalence of latent TB among medical staffs that works in negative-pressure isolation ward against natural ventilation isolation ward at Kandou General Hospital Manado. **Methods:** An cross-sectional study involving 20 medical personnel that have worked for more than 6 months in negative -pressure isolation ward and natural ventilation isolation ward at Kandou General Hospital Manado, North Celebes, Indonesia. Exclusion criteria were history of TB disease or TB latent, immunodeficiency and long term steroid uses. Fischer exact test and regression analysis was used to evaluate the differences between variables. **Results :** There were 7 medical personnel (35%) from the negative-pressure isolation ward compared to 11 medical personnel (55%) in natural ventilation isolation ward that were positive for Interferon Gamma Release Assay (IGRA). There were no significance differences between the type of isolation room and the prevalence of latent tuberculosis (p= 0.341). There were also no significant correlations between room type (p = 0.633), work duration (p = 0.181), and the prevalence of latent TB (R^2^ = 0.06). **Conclusion :** There is no significant difference between latent TB prevalence among medical presonnel in negative- pressure isolation room and natural ventilation isolation room. Natural ventilation room could be used as an alternative to negative-pressure isolation room.

